# Effect of Early and Late Interventions with Dietary Oils on Vascular and Neural Complications in a Type 2 Diabetic Rat Model

**DOI:** 10.1155/2019/5020465

**Published:** 2019-08-07

**Authors:** Lawrence Coppey, Eric Davidson, Hanna Shevalye, Alexander Obrosov, Mark Yorek

**Affiliations:** ^1^Department of Internal Medicine, University of Iowa, Iowa City, IA, USA; ^2^Department of Veterans Affairs Iowa City Health Care System, Iowa City, IA, USA; ^3^Veterans Affairs Center for the Prevention and Treatment of Visual Loss, Iowa City, IA, USA; ^4^Fraternal Order of Eagles Diabetes Research Center, University of Iowa, Iowa City, IA, USA

## Abstract

**Aims:**

Determine the effect of dietary oils enriched in different mono- or polyunsaturated fatty acids, i.e., olive oil (18 : 1, oleic acid), safflower oil (18 : 2 n-6, linoleic acid), flaxseed oil (18 : 3 n-3, alpha linolenic acid), evening primrose oil (18 : 3 n-6, gamma linolenic acid), or menhaden oil (20:5/22 : 6 n-3 eicosapentaenoic/docosahexaenoic acids), on vascular and neural complications in high-fat-fed low-dose streptozotocin-treated Sprague-Dawley rats, an animal model for late-stage type 2 diabetes.

**Materials and Methods:**

Rats were fed a high-fat diet (45% kcal as fat primarily derived from lard) for 8 weeks and then treated with a low dose of streptozotocin (30 mg/kg) in order to induce hyperglycemia. After an additional 8 (early intervention) or 20 (late intervention) weeks, the different groups of rats were fed diets with 1/2 of the kcal of fat derived from lard replaced by the different dietary oils. In addition, a control group fed a standard diet (4.25% kcal as fat) and a diabetic group maintained on the high-fat diet were maintained. The treatment period was approximately 16 weeks. The endpoints evaluated included vascular reactivity of epineurial arterioles, motor and sensory nerve conduction velocity, thermal and corneal sensitivity, and innervation of sensory nerves in the cornea and skin.

**Results:**

Our findings show that menhaden and flaxseed oil provided the greatest benefit for correcting peripheral nerve damage caused by diabetes, whereas enriching the high-fat diet with menhaden oil provided the most benefit to acetylcholine-mediated vascular relaxation of epineurial arterioles of the sciatic nerve. Enriching the diets with fatty acids derived from the other oils provided none to partial improvements.

**Conclusions:**

These studies imply that long-chain n-6 and n-3 polyunsaturated fatty acids could be an effective treatment for diabetic peripheral neuropathy with n-3 polyunsaturated fatty acids derived from fish oil being the most effective.

## 1. Introduction

Peripheral neuropathy is the most common complication of diabetes affecting about 50% of patients [1]. There is no treatment for peripheral neuropathy other than good glycemic control that is primarily only effective in patients with type 1 diabetes [1]. Since symptoms of peripheral neuropathy include pain, numbness, paresthesia, and ulcerations in the extremities that can lead to amputations, finding a treatment to improve the quality of life is vital.

Lipids have been shown to play an important role in complications associated with metabolic syndrome and type 2 diabetes [1, 2]. Recently, in a similar study, we have shown that treating diet-induced obese rats, an animal model of prediabetes, after vascular and nerve pathology had developed, with oils enriched in oleic acid or linoleic acid provided no improvement in vascular or neural function. In that study, we also found that treating diet-induced obese rats with either *γ*- or *α*-linolenic acids, derived from evening primrose oil or flaxseed oil, respectively, provided a moderate benefit. However, we found that treating diet-induced obese rats with menhaden oil enriched in eicosapentaenoic and docosahexaenoic acids provided significant improvement in both vascular and neural functions [3]. The purpose of the present study was to gain a better understanding of the role of these same dietary lipids on vascular relaxation of arterioles that provide circulation to the sciatic nerve and peripheral neuropathy in an animal model of type 2 diabetes. Sprague-Dawley rats treated with a high-fat diet as done in our previous study do not become hyperglycemic [3]. Therefore, for this study, we used Sprague-Dawley rats that were fed a high-fat diet for 8 weeks followed by a low dose of streptozotocin [4]. This approach creates a rat model of late-stage type 2 diabetes [4]. After 8 or 20 weeks of nontreated hyperglycemia, these rats were fed a high-fat diet enriched in oleic acid (olive oil; 18 : 1, n-9), linoleic acid (safflower oil; 18 : 2, n-6), *γ*-linolenic acid (evening primrose oil; 18 : 3, n-6), *α*-linolenic acid (flaxseed oil; 18 : 3, n-3), or eicosapentaenoic/docosahexaenoic acids (menhaden oil; 20 : 5 and 22 : 6, n-3).

## 2. Materials and Methods

### 2.1. Animals, Diets, and Experimental Design

Male Sprague-Dawley (Harlan Sprague Dawley, Indianapolis, IN) rats 10-11 weeks of age were housed in a certified animal care facility, and food and water were provided ad libitum. All institutional and NIH guidelines for use of animals were followed. These studies were approved by the University of Iowa Animal Care and Use Committee (# 5071450). At 12 weeks of age, rats were divided into 7 groups. One group was designated as the control group and remained on the standard diet (Envigo Teklad, #7001, Madison, WI), which contained 25% protein, 4% fat, and 40% carbohydrate. The other 6 groups of rats were placed on a high-fat diet for 8 weeks (D12451; Research Diets, New Brunswick, NJ). The high-fat diet contained 24% protein, 24% fat, and 41% carbohydrate, and 45% of the kcal in this diet is derived from fat. The primary source of the increased fat content in the diet was lard. Afterwards, these rats were treated with a low dose of streptozotocin (30 mg/kg) to induce hyperglycemia [4]. Following 8 weeks (early intervention) or 20 weeks (late intervention) of untreated hyperglycemia, 5 of the 6 groups of diabetic rats were placed on high-fat diets with 1/2 kcal of fat derived from lard replaced with olive oil (D16030902), safflower oil (D16030903), flaxseed oil (D16030904), evening primrose oil (D16030905), or menhaden oil (D16021504). The remaining group of diabetic rats was maintained on the original high-fat diet (D12451) while the control rats remained on the standard diet for the duration of the study. These diets were prepared by Research Diets, and the treatment period was approximately 16 weeks. The olive, safflower, flaxseed, and menhaden oils were provided by Research Diets. Evening primrose oil was purchased from Starwest Botanicals Inc. (Sacramento, CA) and shipped to Research Diets for production of D16030905. The fatty acid composition of each of these diets is presented in Supplemental Table 1.

### 2.2. Endpoints Related to Nerve Function and Vascular Reactivity

As in all our studies, multiple of endpoints related to neural function were determined employing methodology common to our laboratory and detailed information can be found in these references [5–7]. Moreover, the endpoints examined in this study were the same as the endpoints evaluated in our study of the effect of dietary lipids on vascular and neural dysfunction in diet-induced obese rats [3]. The endpoints examined included thermal nociceptive latency of the hindpaws and cornea sensitivity in unanesthetized rats. Motor and sensory nerve conduction velocity and density of corneal nerves by corneal confocal microscopy were determined in rats anesthetized with sodium pentobarbital (50 mg/kg, i.p., Abbott Laboratories, North Chicago, IL). Following these procedures, the anesthetized rats were euthanized by exsanguination via cardiac puncture and tissues were harvested to determine intraepidermal nerve fiber density of the hindpaw, vascular reactivity of epineurial arterioles of the sciatic nerve to acetylcholine and calcitonin gene-related peptide, and liver steatosis as previously described.

### 2.3. Fatty Acid Composition

Fatty acid composition of the diets, serum, and the liver were measured after the lipid fraction was extracted using chloroform/methanol, followed by transesterification in 14% boron trifluoride in methanol and extraction of the fatty acid methyl esters into heptane. The fatty acids were then separated by gas-liquid chromatography [8]. Individual fatty acids peak as % of total fatty acids present were identified by comparison to known fatty acid standards.

### 2.4. Physiological Markers

Nonfasting blood glucose was determined with Aviva Accu-Chek strips. Serum was collected under nonfasting conditions for determining the levels of free fatty acid, triglyceride, and free cholesterol using commercial kits and provided instructions from Roche Diagnostics, Mannheim, Germany; Sigma Chemical Co., and St. Louis, MO; Bio Vision, Mountain View, CA, respectively. Serum was also used to determine thiobarbituric acid-reactive substances as previously described [7].

### 2.5. Data Analysis

Results are presented as mean ± SEM. Comparisons between the groups were conducted using one-way ANOVA and Bonferroni posttest comparison (Prism software; GraphPad, San Diego, CA). Concentration response curves for acetylcholine and calcitonin gene-related peptide were compared using a two-way repeated measures analysis of variance with an autoregressive covariance structure using proc mixed program of SAS data for area under the curve which was also determined for each concentration curve using Prism software and values included in the figure legends [5–7]. A *p* value of less than 0.05 was considered significant.

## 3. Results

### 3.1. Weight and Blood Glucose

Tables 1 and 2 provide data on the starting and final weights and nonfasting blood glucose levels of rats entered into the early (Table 1) or late (Table 2) intervention protocols. At the beginning of the two study protocols, all rats weighed the same. At the end of the early intervention protocol, diabetic rats treated with olive oil, evening primrose oil, or menhaden oil weighed significantly less than control rats. For the late intervention protocol, there was no significant difference in the final weight of the rats. In both protocols, nonfasting blood glucose levels were significantly increased in diabetic rats and were not influenced by the different lipid-enriched diets. The amount of diet consumed was examined in weekly intervals, and diabetic rats generally ate about 15% more chow than control rats, and the lipid composition of the high-fat diets did not significantly affect this pattern (data not shown).

### 3.2. Serum Lipid and Liver Steatosis

Serum free fatty acid levels were significantly elevated in high-fat diet-fed diabetic rats after 24 weeks of diabetes (Table 1) and to a greater extent after 36 weeks (Table 2). With early intervention, serum free fatty acid levels at the end of the study were not increased in diabetic rats receiving the high-fat diet enriched with safflower oil or flaxseed oil. However, in the late intervention group, serum free fatty acid levels were significantly increased in all diabetic rats independent of dietary oil enrichment at the end of the study. Serum triglyceride levels were increased in all diabetic rats at the end of the early or late intervention study with a greater increase occurring in rats with 36 weeks of hyperglycemia (Table 2). There was no significant effect of dietary oils on serum triglyceride levels in diabetic rats. Serum cholesterol levels were increased in high-fat-fed diabetic rats. Early intervention with a high-fat diet enriched with safflower oil or flaxseed oil lowered cholesterol levels. Late intervention of diabetic rats with a high-fat diet enriched with evening primrose oil or flaxseed oil lowered cholesterol levels. Serum thiobarbituric acid substances, a marker of oxidative stress, were significantly increased in all diabetic rats and not altered after changing the dietary lipid content of the high-fat diet.

Recent studies have demonstrated an association between nonalcoholic fatty liver disease and diabetic neuropathy [9, 10]. Liver steatosis was increased in high-fat-fed diabetic rats after 24 (Table 1) or 36 (Table 2) weeks of hyperglycemia. Enriching the diets of high-fat-fed diabetic rats with oils containing mono- or polyunsaturated fatty acids partially lowered the fatty acid content of the liver compared to untreated high-fat-fed diabetic rats, but in all cases, liver steatosis remained significantly increased compared to control rats.

### 3.3. Fatty Acid Composition of Serum and the Liver

Supplemental Tables 2 and 3 present data for the fatty acid composition of serum following early and late dietary interventions, respectively. In serum, the primary fatty acid enriched in each of the oils was generally increased compared to control and/or high-fat-fed diabetic rats. For the early intervention (Supplemental Table 2), the increase was significant for each of the primary fatty acids derived from each of the dietary oils, and for the late intervention (Supplemental Table 3), a significant increase in the primary fatty acid in serum was achieved with only flaxseed oil and menhaden oil.

Supplemental Tables 4 and 5 present data for the fatty acid composition of the liver following early and late interventions, respectively. The changes that occurred in the fatty acid composition of the liver were similar to that observed in serum, and generally, there was an increase in the primary fatty acid derived from each of the dietary oils in the liver. The increase was only consistently significant for flaxseed oil and menhaden oil. Interestingly, the content of arachidonic acid was significantly decreased in the liver of diabetic rats treated with high-fat diets enriched in flaxseed oil or menhaden oil compared to control or untreated diabetic rats. Following the early intervention, the fatty acid unsaturation index was significantly increased in serum and liver of diabetic rats treated with a high-fat diet enriched in menhaden oil (Supplemental Table 6). In the late intervention study, the fatty acid unsaturation index was significantly increased in serum of diabetic rats treated with a high-fat diet enriched in menhaden oil and in the liver of diabetic rats treated with a high-fat diet enriched in either flaxseed oil or menhaden oil (Supplemental Table 7).

Recent studies have suggested that reducing the n-6 to n-3 fatty acid ratio improves inflammatory conditions [11, 12]. In this study, we found that treating high-fat-fed diabetic rats following an early or late intervention with a high-fat diet enriched in menhaden oil or to a lesser extent flaxseed oil significantly reduces the n-6 to n-3 fatty acid ratio in serum and the liver (Supplemental Table 8 or 9, respectively).

### 3.4. Neural and Vascular Endpoints

**Figure 1:**
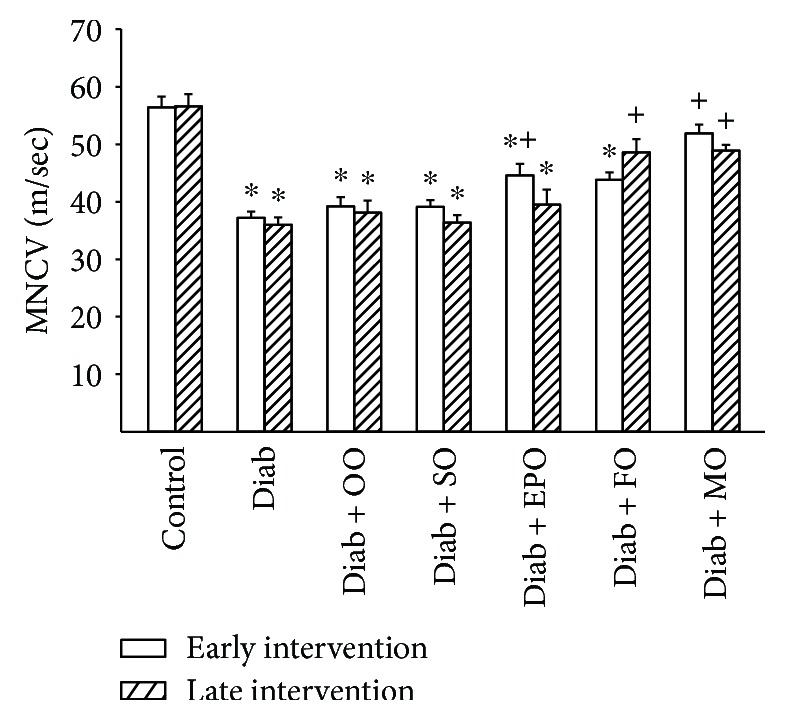
Effect of dietary oils on motor nerve conduction velocity in high-fat-fed diabetic Sprague-Dawley rats. Motor nerve conduction velocity was examined after early (open bars) and late (hatched bars) interventions as described in Materials and Methods. Data are presented as the mean ± SEM in m/sec. The number of rats in each group was the same as shown in Tables 1 and 2, for the early and late intervention periods, respectively. Motor nerve conduction velocities for control and diabetic rats at the beginning of the early intervention treatment were 52.5 ± 1.7 and 37.4 ± 1.7 m/sec, respectively. ^∗^ *P* < 0.05 compared to control rats; ^+^ *P* < 0.05 compared to diabetic rats. OO: olive oil; SO: safflower oil; EPO: evening primrose oil; FO: flaxseed oil; MO: menhaden oil.

**Figure 2:**
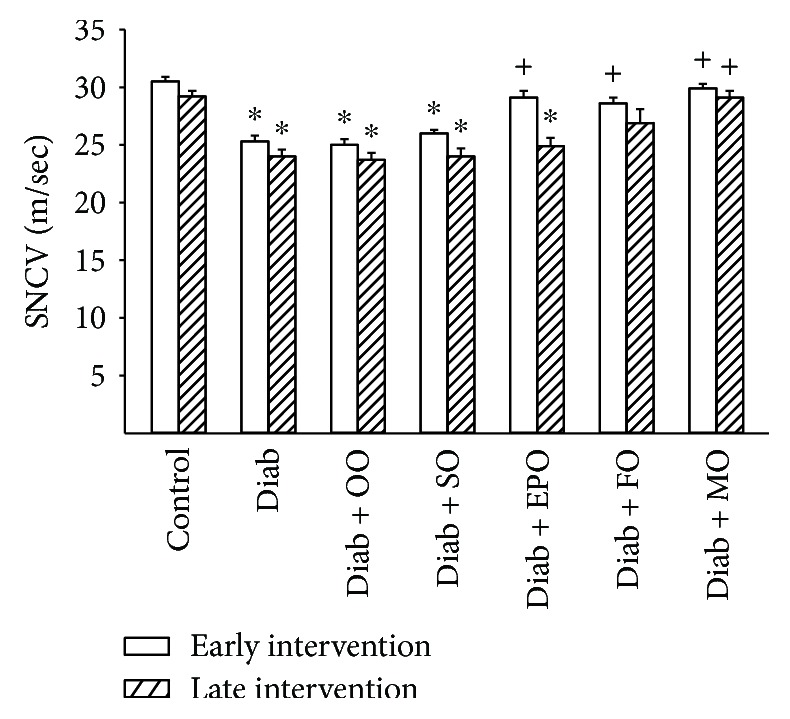
Effect of dietary oils on sensory nerve conduction velocity in high-fat-fed diabetic Sprague-Dawley rats. Sensory nerve conduction velocity was examined after early (open bars) and late (hatched bars) interventions as described in Materials and Methods. Data are presented as the mean ± SEM in m/sec. The number of rats in each group was the same as shown in Tables 1 and 2, for the early and late intervention periods, respectively. Sensory nerve conduction velocities for control and diabetic rats at the beginning of the early intervention treatment were 31.9 ± 1.1 and 26.9 ± 0.7 m/sec, respectively. ^∗^ *P* < 0.05 compared to control rats; ^+^ *P* < 0.05 compared to diabetic rats. OO: olive oil; SO: safflower oil; EPO: evening primrose oil; FO: flaxseed oil; MO: menhaden oil.

Data in Figures 1 and 2 demonstrate that motor and sensory nerve conduction velocity is decreased in Sprague-Dawley rats modeling type 2 diabetes with chronic hyperglycemia. Early intervention with evening primrose oil of the high-fat diet improved both motor and sensory nerve conduction velocities, but this treatment was ineffective in the late intervention protocol. Enriching the high-fat diet with flaxseed oil was also partially effective in improving motor and to a greater extent sensory nerve conduction velocity. However, enriching the high-fat diet early or late with menhaden oil significantly improved both motor and sensory nerve conduction velocities. Enriching the diet of diabetic rats with olive oil or safflower oil provided no improvement in either motor or sensory nerve conduction velocity whether it was given early or late after the onset of hyperglycemia.

**Figure 3:**
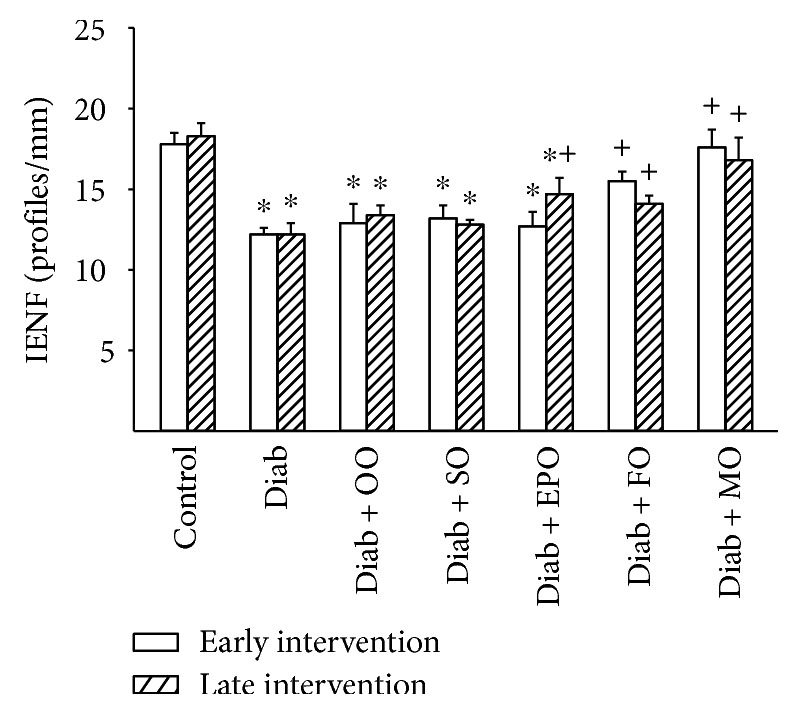
Effect of dietary oils on intraepidermal nerve fiber density in high-fat-fed diabetic Sprague-Dawley rats. Intraepidermal nerve fiber density was examined after early (open bars) and late (hatched bars) interventions as described in Materials and Methods. Data are presented as the mean ± SEM in profiles per mm. The number of rats in each group was the same as shown in Tables 1 and 2, for the early and late intervention periods, respectively. Intraepidermal nerve fiber densities for control and diabetic rats at the beginning of the early intervention treatment were 16.9 ± 1.0 and 13.1 ± 0.6 profiles/mm, respectively. ^∗^ *P* < 0.05 compared to control rats; ^+^ *P* < 0.05 compared to diabetic rats. OO: olive oil; SO: safflower oil; EPO: evening primrose oil; FO: flaxseed oil; MO: menhaden oil.

**Figure 4:**
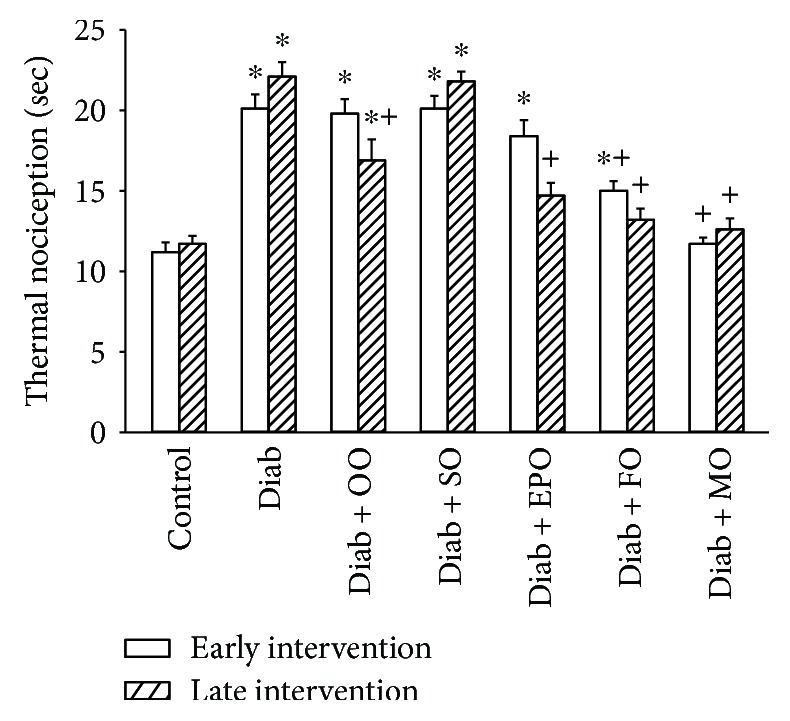
Effect of dietary oils on thermal nociception in high-fat-fed diabetic Sprague-Dawley rats. Thermal nociception was examined after early (open bars) and late (hatched bars) interventions as described in Materials and Methods. Data are presented as the mean ± SEM in sec. The number of rats in each group was the same as shown in Tables 1 and 2, for the early and late intervention periods, respectively. Thermal nociception for control and that for diabetic rats at the beginning of the early intervention treatment were 12.3 ± 0.2 and 20.1 ± 0.9 sec, respectively. ^∗^ *P* < 0.05 compared to control rats; ^+^ *P* < 0.05 compared to diabetic rats. OO: olive oil; SO: safflower oil; EPO: evening primrose oil; FO: flaxseed oil; MO: menhaden oil.

Thermal nociception and density of sensory nerve fibers in the skin of the hindpaw are common neuro-related endpoints examined in preclinical studies of diabetes in rodents. Data in Figures 3 and 4 demonstrate that in a rat modeling type 2 diabetes, there is a significant decrease in nerve fibers in the skin and latent response to a thermal stimulus. Treating diabetic rats early or late with olive oil or safflower oil had minimal to no effect on these two endpoints. Treatment (late intervention) with evening primrose oil partially improved the density of intraepidermal nerve fibers and thermal nociception. Treating diabetic rats fed a high-fat diet by replacing 1/2 of the kcal of the high-fat diet with flaxseed oil or to a greater extent menhaden oil significantly improved both intraepidermal nerve fiber density and thermal nociception.

**Figure 5:**
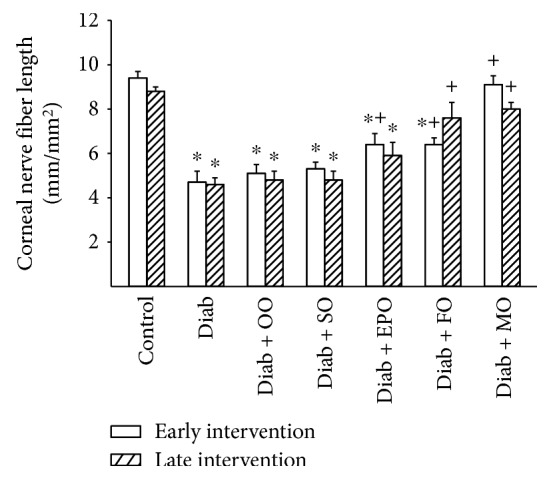
Effect of dietary oils on cornea nerve fiber length in high-fat-fed diabetic Sprague-Dawley rats. Innervation of the subepithelial layer of the cornea was determined after early (open bars) and late (hatched bars) interventions by using corneal confocal microscopy as described in Materials and Methods. Data are presented as the mean ± SEM for innervation of the cornea in mm/mm^2^. The number of rats in each group was the same as shown in Tables 1 and 2, for the early and late intervention periods, respectively. Cornea nerve fiber lengths for control and diabetic rats at the beginning of the early intervention treatment were 8.4 ± 0.4 and 4.4 ± 0.5 mm/mm^2^, respectively. ^∗^ *P* < 0.05 compared to control rats; ^+^ *P* < 0.05 compared to diabetic rats. OO: olive oil; SO: safflower oil; EPO: evening primrose oil; FO: flaxseed oil; MO: menhaden oil.

**Figure 6:**
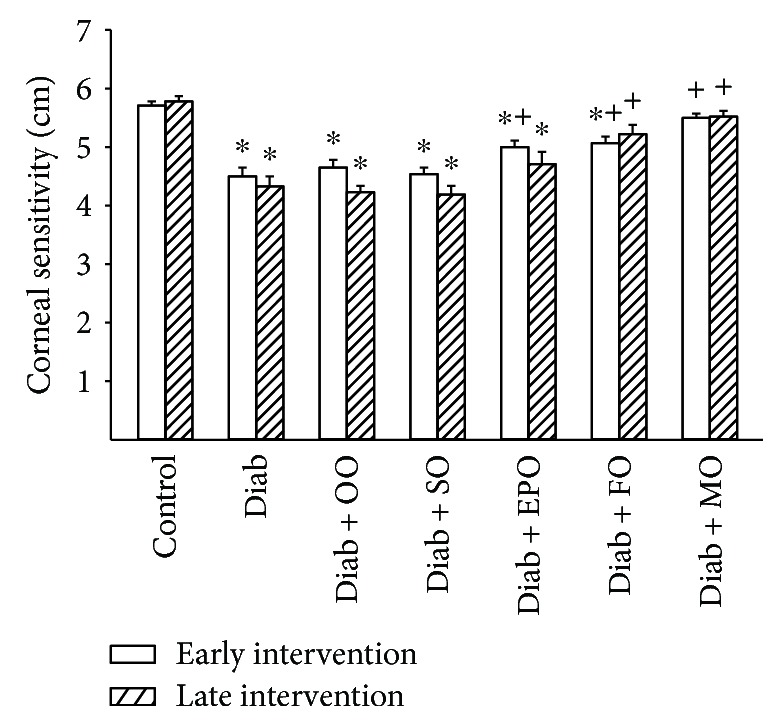
Effect of dietary oils on cornea sensitivity in high-fat-fed diabetic Sprague-Dawley rats. Corneal sensitivity was determined after early (open bars) and late (hatched bars) interventions by using Cochet-Bonnet filament esthesiometer as described in Materials and Methods. Data are presented as the mean ± SEM for corneal sensitivity in cm. The number of rats in each group was the same as shown in Tables 1 and 2, for the early and late intervention periods, respectively. Corneal sensitivities for control and diabetic rats at the beginning of the early intervention treatment were 5.9 ± 0.1 and 5.0 ± 0.2 cm, respectively. ^∗^ *P* < 0.05 compared to control rats; ^+^ *P* < 0.05 compared to diabetic rats. OO: olive oil; SO: safflower oil; EPO: evening primrose oil; FO: flaxseed oil; MO: menhaden oil.

Recently, examination of corneal nerve fiber density of the subepithelial layer and cornea sensitivity has been promoted as a surrogate marker for progression of diabetic neuropathy in human subjects [13]. Data in Figures 5 and 6 demonstrate that chronic hyperglycemia causes a decrease in corneal nerves and abnormal cornea sensitivity. Treating high-fat-fed diabetic rats early or late after the onset of hyperglycemia with high-fat diets enriched with olive oil or safflower oil did not correct cornea nerve fiber density or corneal sensitivity. Early intervention with evening primrose oil was partially effective in improving cornea nerve fiber density and sensitivity. Early and late interventions with flaxseed oil were also partially effective. However, early or late dietary intervention of diabetic rats with menhaden oil completely restored both cornea nerve density and sensitivity.

**Figure 7:**
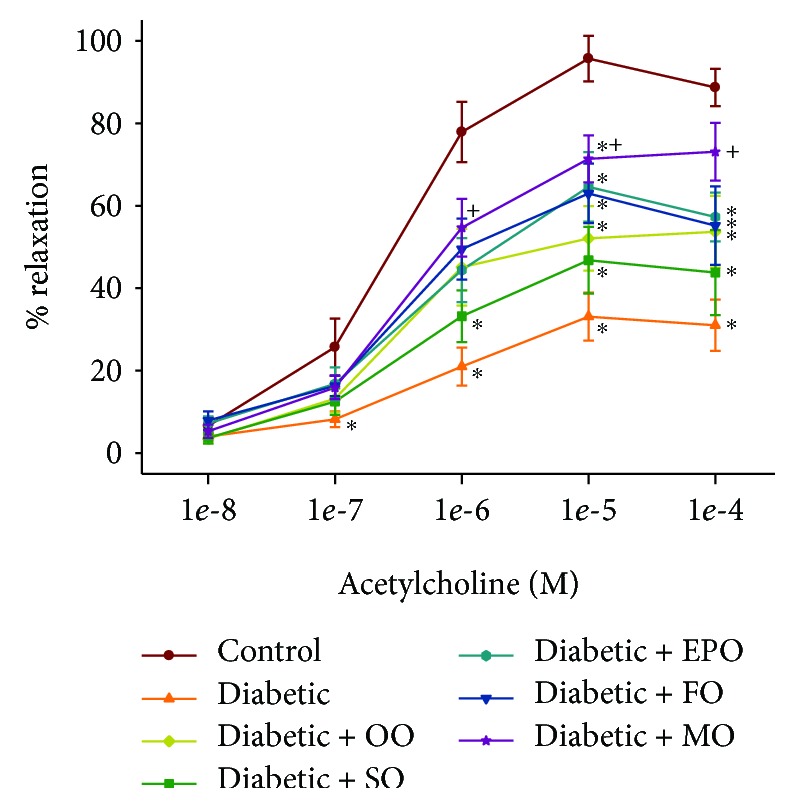
Effect of early intervention with dietary oils on vascular relaxation by acetylcholine in epineurial arterioles of the sciatic nerve in high-fat-fed diabetic Sprague-Dawley rats. Pressurized arterioles (40 mm Hg and ranging from 60 to 100 *μ*m luminal diameters) were constricted with phenylephrine (30-50%), and incremental doses of acetylcholine were added to the bathing solution while recording the steady-state vessel diameter. Data are presented as the mean of % relaxation ± SEM. The number of rats in each group was the same as shown in Table 1. Using area under the curve to compare the effect of diabetes and treatments to relaxation by acetylcholine: control vs. diabetic, *P* < 0.001; control vs. diabetic+OO, *P* < 0.02; control vs. diabetic+SO, *P* < 0.001; control vs. diabetic+EPO, *P* < 0.005; control vs. diabetic+FO, *P* < 0.05; diabetic vs. diabetic+MO, *P* < 0.02. OO: olive oil; SO: safflower oil; EPO: evening primrose oil; FO: flaxseed oil; MO: menhaden oil.

**Figure 8:**
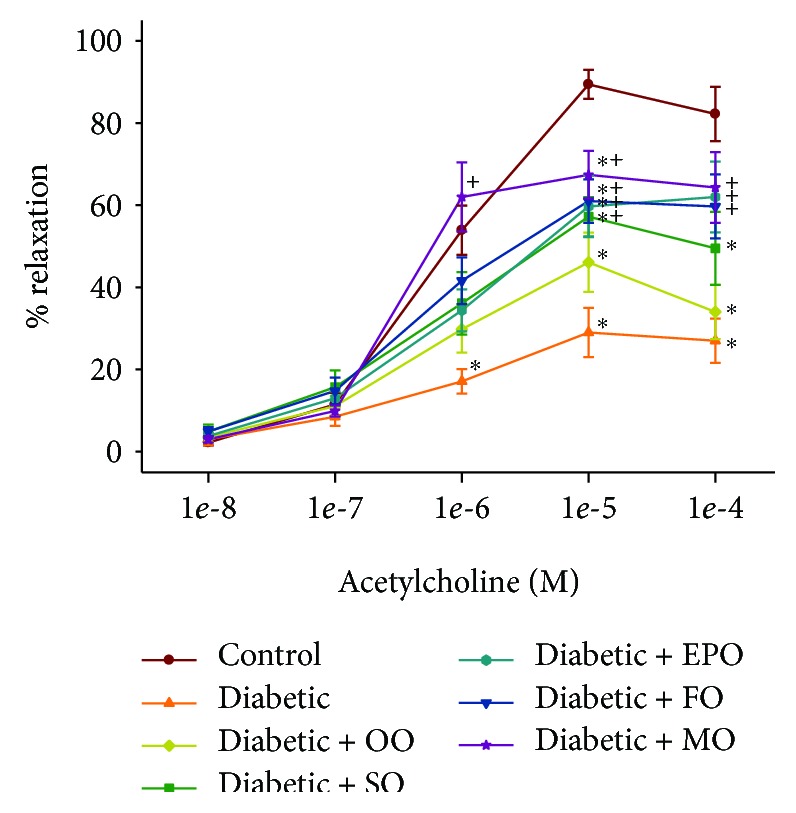
Effect of late intervention with dietary oils on vascular relaxation by acetylcholine in epineurial arterioles of the sciatic nerve in high-fat-fed diabetic Sprague-Dawley rats. Pressurized arterioles (40 mm Hg and ranging from 60 to 100 *μ*m luminal diameters) were constricted with phenylephrine (30-50%), and incremental doses of acetylcholine were added to the bathing solution while recording the steady-state vessel diameter. Data are presented as the mean of % relaxation ± SEM. The number of rats in each group was the same as shown in Table 2. Using area under the curve to compare the effect of diabetes and treatments to relaxation by acetylcholine: control vs. diabetic, *P* < 0.001; control vs. diabetic+OO, *P* < 0.01; diabetic vs. diabetic+FO, *P* < 0.05; diabetic vs. diabetic+MO, *P* < 0.005. OO: olive oil; SO: safflower oil; EPO: evening primrose oil; FO: flaxseed oil; MO: menhaden oil.

**Figure 9:**
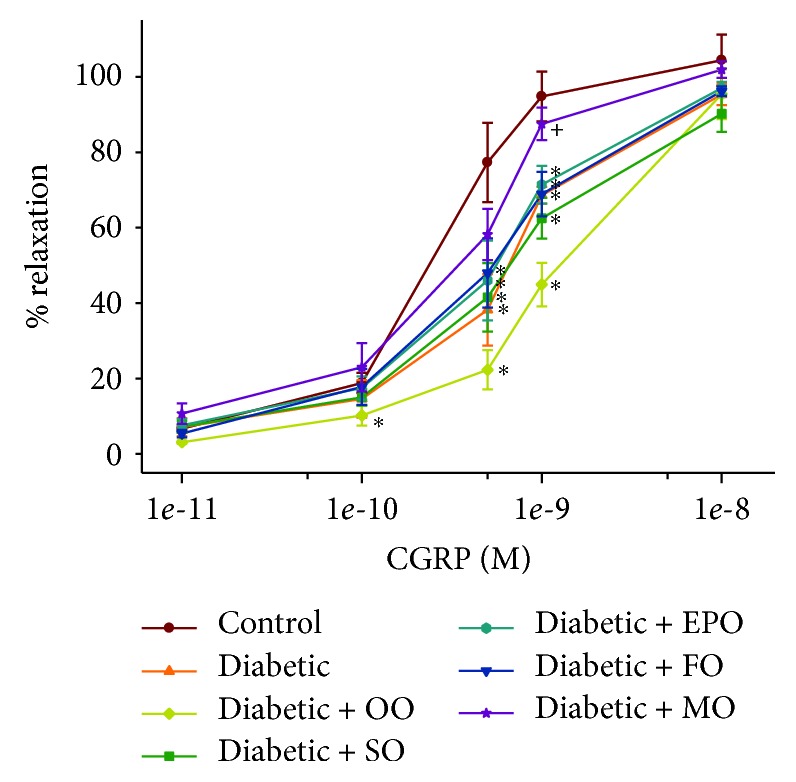
Effect of early intervention with dietary oils on vascular relaxation by calcitonin gene-related peptide in epineurial arterioles of the sciatic nerve in high-fat-fed diabetic Sprague-Dawley rats. Pressurized arterioles (40 mm Hg and ranging from 60 to 100 *μ*m luminal diameters) were constricted with phenylephrine (30-50%), and incremental doses of calcitonin gene-related peptide were added to the bathing solution while recording the steady-state vessel diameter. Data are presented as the mean of % relaxation ± SEM. The number of rats in each group was the same as shown in Table 1. Using area under the curve to compare the effect of diabetes and treatments to relaxation by calcitonin gene-related peptide: control vs. diabetic, *P* < 0.05; control vs. diabetic+OO, *P* < 0.01; control vs. diabetic+SO, *P* < 0.05; control vs. diabetic+EPO, *P* < 0.05; control vs. diabetic+FO, *P* < 0.05; diabetic vs. diabetic+MO, *P* < 0.05. OO: olive oil; SO: safflower oil; EPO: evening primrose oil; FO: flaxseed oil; MO: menhaden oil.

**Figure 10:**
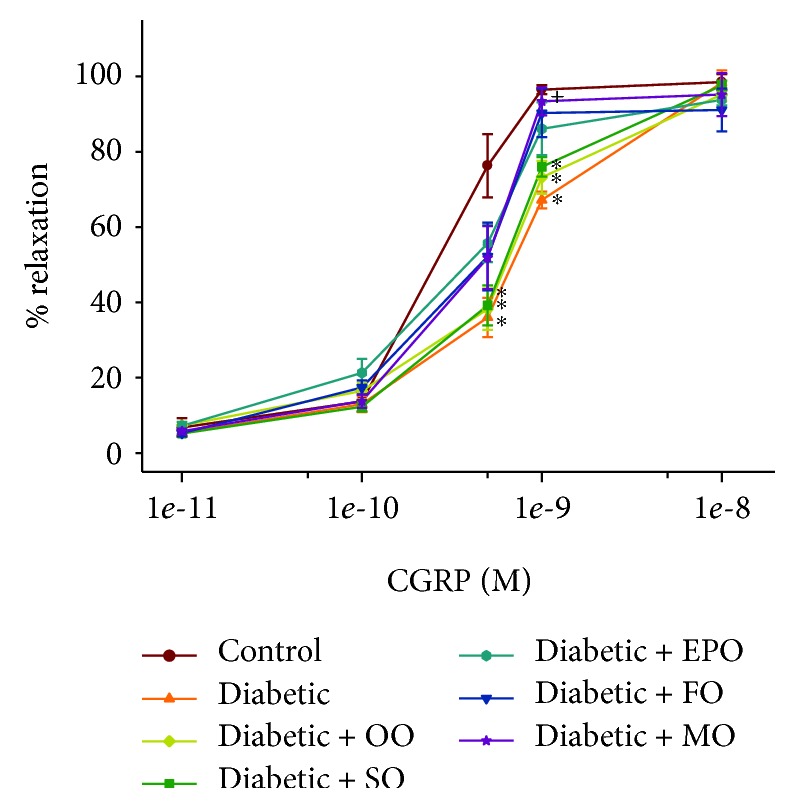
Effect of late intervention with dietary oils on vascular relaxation by calcitonin gene-related peptide in epineurial arterioles of the sciatic nerve in high-fat-fed diabetic Sprague-Dawley rats. Pressurized arterioles (40 mm Hg and ranging from 60 to 100 *μ*m luminal diameters) were constricted with phenylephrine (30-50%), and incremental doses of calcitonin gene-related peptide were added to the bathing solution while recording the steady-state vessel diameter. Data are presented as the mean of % relaxation ± SEM. The number of rats in each group was the same as shown in Table 2. Using area under the curve to compare the effect of diabetes and treatments to relaxation by calcitonin gene-related peptide: control vs. diabetic, *P* < 0.05; control vs. diabetic+OO, *P* < 0.05; control vs. diabetic+SO, *P* < 0.05; diabetic vs. diabetic+MO, *P* < 0.05. OO: olive oil; SO: safflower oil; EPO: evening primrose oil; FO: flaxseed oil; MO: menhaden oil.

We have previously demonstrated that vasodilation of epineurial arterioles, blood vessels that provide circulation to the sciatic nerve, to acetylcholine is decreased prior to slowing of nerve conduction velocity in diabetic rats, and after 8 weeks of chronic hyperglycemia vasodilation to calcitonin, gene-related peptide is also decreased [14, 15]. At the time of early intervention, vascular relaxation of epineurial arterioles to acetylcholine and calcitonin gene-related peptide was significantly decreased (Supplemental Figure 1). Data in Figures 7 and 8 confirm that acetylcholine-mediated vascular relaxation is impaired in rats modeling type 2 diabetes after 24 (Figure 7) or 36 (Figure 8) weeks of chronic and untreated hyperglycemia. Treating high-fat-fed diabetic rats by enriching their diets with olive oil or safflower oil was the least effective in improving vascular reactivity to acetylcholine regardless of early or late intervention. Treating diabetic rats by dietary enrichment with either evening primrose oil or flaxseed oil was partially effective in improving vascular relaxation to acetylcholine. However, the greatest benefit to acetylcholine-mediated vascular relaxation was observed when diabetic rats were treated early or late with a diet enriched with menhaden oil. Data in Figures 9 and 10 demonstrate that chronic hyperglycemia for 24 (Figure 9) or 36 weeks (Figure 10) causes a significant decrease in vascular relaxation of epineurial arterioles to calcitonin gene-related peptide. Early intervention by replacing 1/2 of the kcal of fat in the high-fat diet with olive oil, safflower oil, evening primrose oil, or flaxseed oil provided no observable improvement in vascular relaxation by diabetic rats to calcitonin gene-related peptide (Figure 9). However, with early intervention, improvement in vascular relaxation to calcitonin gene-related peptide was observed when the diet of high-fat-fed diabetic rats was enriched with menhaden oil. Late intervention of high-fat-fed diabetic rats with diets enriched with menhaden oil as well as with evening primrose oil or flaxseed oil provided partial improvement in vascular relaxation to calcitonin gene-related peptide (Figure 10). In contrast, late intervention of diabetic rats with a diet enriched with olive oil or safflower oil provided no improvement in vascular relaxation to calcitonin gene-related peptide.

## 4. Discussion

Limited information is available on the effect that mono- or polyunsaturated fatty acids of the omega-6 or omega-3 classes may have on vascular and neural defects on rats modeling late-stage type 2 diabetes. To address this question, we investigated the effect of 16-week treatment of high-fat-fed diabetic rats following early (8 weeks post hyperglycemia) or late (20 weeks post hyperglycemia) intervention of hyperglycemia/high-fat diet with 1/2 of the kcal of fat derived from lard replaced with olive oil (enriched in oleic acid, 18 : 1 n-9 mono-unsaturated fatty acid), safflower oil (enriched in linoleic acid, 18 : 2 n-6 polyunsaturated fatty acid), evening primrose oil (enriched in *γ*-linolenic acid, 18 : 3 n-6 polyunsaturated fatty acid), flaxseed oil (enriched in *α*-linolenic acid, 18 : 3 n-3 polyunsaturated fatty acid), or menhaden oil (enriched in eicosapentaenoic and docosahexaenoic fatty acid, 20 : 5 and 22 : 6 n-3 polyunsaturated fatty acid). At the time of the early intervention treatment, diabetic rats had impaired neural function as determined by slowing of motor and sensory nerve conduction velocity, decreased density of intraepidermal nerve fibers and corneal nerve fiber length, and decreased thermal nociception and cornea sensitivity. In addition, at the time of early intervention, vascular relaxation by epineurial arterioles to acetylcholine and calcitonin gene-related peptide was decreased. At the time of late intervention, vascular and neuropathy endpoints were not significantly more impaired than at the time of early intervention. This is consistent with our earlier longitudinal studies that have shown a progressive decline in vascular and neural functions from 2 to 8 weeks post hyperglycemia after which the pathology in this late-stage type 2 diabetic rat model stabilizes. Nonetheless, we deemed it important to examine both early and late phases of diabetes to determine whether vascular and neural endpoints are reversible after prolonged hyperglycemia.

Treating diabetic rats with a high-fat diet enriched with oleic acid (olive oil) except for some improvement in thermal nociception with late intervention had no significant benefit on the neural endpoints and vascular relaxation to acetylcholine, and calcitonin gene-related peptide remained significantly impaired. We obtained similar results when we enriched diets of diet-induced obese rats with olive oil [3]. The serum lipid profile after early and late interventions was not changed compared to high-fat-fed diabetic rats nor was the fatty acid unsaturation index or n-6 to n-3 fatty acid ratio of serum or the liver. In addition, liver steatosis and a serum marker of oxidative stress remained elevated in diabetic rats treated with olive oil. Nutritional intake of olive oil is the key component of the Mediterranean diet that has been associated with the prevention and management of many chronic diseases including type 2 diabetes [16, 17]. However, the Mediterranean diet is rich in extra virgin olive oil that is enriched with vitamins and polyphenols that have been shown to have antioxidative and anti-inflammatory properties [18–20]. Extra virgin olive oil has been shown to ameliorate nonalcoholic steatohepatitis in high-fat Western diet-treated mice [21]. It is believed that reducing saturated fat intake through increasing consumption of monounsaturated fatty acids or polyunsaturated fatty acids will reduce obesity and conditions associated with metabolic syndrome [22]. In our studies, replacing 1/2 of the kcal derived from saturated fatty acids from lard with olive oil was found to be beneficial in reducing hepatic steatosis and thermal nociception after late intervention; however, improvements appeared marginal and likely not of physiological significance. This lack of effect could be due to the conditions of the study design that even with early intervention, the olive oil-enriched diet was not able to overcome the existing deleterious effects of the high-fat diet/hyperglycemia condition on vascular and neural endpoints prior to treatment and/or the olive oil used in the diet did not have a sufficient concentration of vitamins and polyphenols of extra virgin olive oil.

The rationale for treating diabetes complications including vascular dysfunction and neuropathy with polyunsaturated fatty acids such as gamma linolenic acid is because of the diabetes-induced impairment of delta-6-desaturase [23–25]. Delta-6-desaturase converts linoleic acid into gamma linolenic acid and it is thought that increasing the intake of gamma linolenic acid in diabetes would circumvent this issue. In our studies, treating diabetic rats with a high-fat diet with 1/2 of the kcal derived from safflower oil that is enriched in linoleic acid (18 : 2, n-6 polyunsaturated fatty acid) did not improve vascular or neural endpoints. This is likely due to the inability to convert linoleic acid to gamma linolenic acid. Levels of linoleic acid were increased in the serum and the liver of safflower oil-treated rats, but there was no increase in gamma linolenic acid. Evening primrose oil is enriched in gamma linolenic acid (18 : 3, n-6 polyunsaturated fatty acid). In this study, treating type 2 diabetic rats with a high-fat diet enriched with evening primrose oil partially improved vascular and neural functions. We obtained similar results when diabetic rats were treated with flaxseed oil, which is enriched in alpha linolenic acid (18 : 3, n-3 polyunsaturated fatty acid). The levels of gamma linolenic acid in serum and the liver where only marginally but nonsignificantly increased when diabetic rats were treated with evening primrose oil except for the increase in serum following early intervention (Supplemental Table 2). This result is of interest because treating diabetic rats with evening primrose oil partially improved motor and sensory nerve conduction only with early intervention. It is unknown whether the small increase in serum levels of gamma linolenic acid with early intervention contributed to the marginal increase in nerve conduction velocities in diabetic rats treated with evening primrose oil. The unsaturation index of serum or the liver was not increased in diabetic rats treated with gamma linolenic acid nor was the n-6 to n-3 fatty acid ratio suggesting that these parameters were not predictive of the small improvement in nerve conduction velocities with early intervention of evening primrose oil of diabetic rats. In contrast, levels of alpha linolenic acid were significantly increased in serum and the liver of diabetic rats when treated with flaxseed oil. This is likely because levels of gamma linolenic acid in evening primrose oil compared to levels of alpha linolenic acid in flaxseed oil are much lower (Supplemental Table 1). Nonetheless, evening primrose oil has been widely used as a source of gamma linolenic acid in preclinical studies. In early studies, evening primrose oil was shown to improve nerve conduction velocity in streptozotocin type 1 diabetic rats presumably by improving sciatic nerve blood flow and endoneurial oxygen tension [26, 27]. Treating streptozotocin type 1 diabetic rats with evening primrose oil has also been shown to improve ultrastructural deficits of axons as well as axonal transport and restore Na+/K+ ATPase activity in the sciatic nerve [23, 28, 29]. To the best of our knowledge, no preclinical studies have been performed examining the effect of flaxseed oil on diabetic peripheral neuropathy. However, it has been shown that dietary flaxseed oil ameliorates renal oxidative stress, protein glycation, and inflammation in streptozotocin-nicotinamide-induced diabetic rats, a model for type 2 diabetes [30]. The authors of this study suggested that n-3 polyunsaturated fatty acids may slow progression of diabetic nephropathy. Flaxseed oil has also been shown to attenuate hepatic steatosis and insulin resistance in high-fat-fed mice through improving endoplasmic reticulum stress [31]. In type 2 diabetic rats, flaxseed oil has been shown to alleviate protein glycation and inflammation in the liver [32, 33]. In our studies, we found that treating diabetic rats with flaxseed oil lead to an increase in the levels of eicosapentaenoic acid in serum and liver and a significant decrease in the n-6 to n-3 fatty acid ratio in serum. Overall, there was a trend for late intervention with flaxseed oil to be more efficacious in improving neural outcome measures and vascular relaxation to calcitonin gene-related peptide compared to early intervention. This was surprising since the duration of treatment was the same for both early and late interventions. It is possible that prolonged hyperglycemia may lead to increased sensitivity to exposure of long-chain n-3 polyunsaturated fatty acids. Long-chain n-3 polyunsaturated fatty acids such as eicosapentaenoic acid have been shown to have anti-inflammatory properties. Interestingly, analysis of data from the National Health and Nutrition Examination Survey found that adults with diabetes whose linolenic acid intake was in the highest quintile had lower odds of peripheral neuropathy than adults in the lowest quintile [34]. This analysis is supported by a small clinical double-blind placebo-controlled study done over 25 years ago that demonstrated that treating diabetic patients with distal diabetic polyneuropathy with 360 mg of gamma linolenic acid for 6 months provided significant improvement in neuropathy-related endpoints [35]. Future studies need to carefully evaluate the effect of treatment of diabetes on animal models as well as on human subjects with n-6 and n-3 polyunsaturated fatty acids on lipidomics and inflammatory mediators.

In treating type 2 diabetic rats with mono- or polyunsaturated fatty acids, the greatest benefit toward improving vascular dysfunction and neuronal activity in diabetic rats was observed with menhaden oil that is enriched with eicosapentaenoic and docosahexaenoic acids (20 : 5 and 22 : 6, respectively, n-3 polyunsaturated fatty acids). Diabetic rats treated with menhaden oil had a significant increase in both of these fatty acids in serum and the liver. There was also a significant increase in the unsaturation index in serum and the liver and a significant decrease in the ratio of n-6 to n-3 fatty acids in serum and the liver. Reducing the n-6 to n-3 fatty acid ratio is associated with a decrease in inflammatory stress [11]. The n-3 polyunsaturated fatty acids, eicosapentaenoic and docosahexaenoic acids, are precursors for E and D class resolvins that are credited for the anti-inflammatory properties of these fatty acids [36, 37]. Previously, we have demonstrated that treating streptozotocin diabetic mice with daily injections of resolvins improved neural endpoints and also stimulated nerve filament elongation in vitro [38, 39].

## 5. Conclusions

Our studies have shown that treating a rat model of type 2 diabetes with long-chain n-6 or n-3 polyunsaturated fatty acids following an early or late intervention protocol can improve vascular and neural deficits. Treating diabetic rats with either gamma or alpha linolenic acids, derived from evening primrose oil or flaxseed oil, respectively, provided a moderate benefit. In contrast, treating these rats with menhaden oil enriched in eicosapentaenoic and docosahexaenoic acids provided significant improvement in vascular and neural dysfunction. We previously reported similar results after dietary lipid modification of diet-induced obese rats leading us to conclude that increasing the dietary intake of fish oils may be a potential treatment for vascular and neural complications associated with prediabetes or type 2 diabetes.

## Figures and Tables

**Table 1:** Effect of dietary oils on weight gain, serum lipids, and thiobarbituric acid substances and liver steatosis in type 2 diabetic Sprague-Dawley rats: early intervention.

Determination	Control (12)	Diabetic (14)	Diabetic+olive oil (12)	Diabetic+safflower oil (13)	Diabetic+evening primrose oil (12)	Diabetic+flaxseed oil (13)	Diabetic+menhaden oil (14)
Start weight (g)	362 ± 7	348 ± 5	352 ± 4	349 ± 4	349 ± 4	341 ± 3	349 ± 5
End weight (g)	538 ± 18	452 ± 22	459 ± 17^a^	479 ± 17	453 ± 12^a^	470 ± 18	454 ± 12^a^
Blood glucose (mg/dl)	138 ± 7	404 ± 27^a^	463 ± 28^a^	423 ± 28^a^	437 ± 26^a^	464 ± 25^a^	486 ± 20^a^
Serum free fatty acid (mmol/l)	0.09 ± 0.01	0.22 ± 0.04^a^	0.28 ± 0.04^a^	0.14 ± 0.03	0.48 ± 0.07^a,b^	0.15 ± 0.01	0.33 ± 0.02^a^
Serum triglycerides (mg/dl)	19 ± 3	183 ± 29^a^	281 ± 96^a^	198 ± 65^a^	238 ± 57^a^	130 ± 31^a^	176 ± 53^a^
Serum cholesterol (mg/ml)	4.2 ± 0.2	9.7 ± 0.9^a^	13.5 ± 2.2^a^	7.6 ± 0.7	8.6 ± 1.0^a^	7.4 ± 0.6	9.6 ± 1.2^a^
Serum thiobarbituric acid substances (*μ*g/ml)	0.55 ± 0.08	1.15 ± 0.11^a^	1.05 ± 0.07^a^	1.02 ± 0.10^a^	1.02 ± 0.08^a^	1.15 ± 0.14^a^	1.15 ± 0.14^a^
Liver steatosis (%)	5.1 ± 0.5	40.8 ± 1.3^a^	33.2 ± 1.2^a^	30.8 ± 1.5^a,b^	34.0 ± 1.5^a^	30.1 ± 1.9^a,b^	29.3 ± 1.4^a,b^

Data are presented as the mean ± S.E.M. ^a^ *P* < 0.05 compared to control rats. ^b^ *P* < 0.05 compared to diabetic rats. Parentheses indicate the number of experimental animals.

**Table 2:** Effect of dietary oils on weight gain, serum lipids, and thiobarbituric acid substances and liver steatosis in type 2 diabetic Sprague-Dawley rats: late intervention.

Determination	Control (10)	Diabetic (12)	Diabetic+olive oil (12)	Diabetic+safflower oil (12)	Diabetic+evening primrose oil (12)	Diabetic+flaxseed oil (12)	Diabetic+menhaden oil (12)
Start weight (g)	346 ± 5	358 ± 3	347 ± 6	352 ± 4	349 ± 3	340 ± 6	352 ± 6
End weight (g)	547 ± 21	496 ± 19	506 ± 21	496 ± 15	498 ± 21	514 ± 21	520 ± 20
Blood glucose (mg/dl)	126 ± 4	438 ± 48^a^	431 ± 46^a^	389 ± 46^a^	447 ± 46^a^	361 ± 47^a^	424 ± 48^a^
Serum free fatty acid (mmol/l)	0.13 ± 0.01	0.44 ± 0.08^a^	0.52 ± 0.04^a^	0.54 ± 0.14^a^	0.36 ± 0.08^a^	0.41 ± 0.05^a^	0.60 ± 0.07^a^
Serum triglycerides (mg/dl)	47 ± 12	773 ± 176^a^	1248 ± 270^a^	613 ± 191^a^	394 ± 49^a^	536 ± 134^a^	829 ± 156^a^
Serum cholesterol (mg/ml)	4.7 ± 0.5	9.4 ± 1.3^a^	8.9 ± 1.5^a^	12.6 ± 1.5^a^	7.6 ± 1.0	7.4 ± 0.6	8.6 ± 0.9^a^
Serum thiobarbituric acid substances (*μ*g/ml)	0.42 ± 0.04	1.20 ± 0.13^a^	1.15 ± 0.09^a^	1.13 ± 0.13^a^	1.12 ± 0.08^a^	1.25 ± 0.13^a^	1.25 ± 0.14^a^
Liver steatosis (%)	5.8 ± 0.4	44.4 ± 1.2^a^	36.2 ± 1.2^a,b^	34.1 ± 1.1^a,b^	35.4 ± 1.2^a,b^	33.5 ± 1.5^a,b^	31.8 ± 1.4^a,b^

Data are presented as the mean ± SEM. ^a^ *P* < 0.05 compared to control rats. ^b^ *P* < 0.05 compared to diabetic rats. Parentheses indicate the number of experimental animals.

## Data Availability

The data used to support the findings of this study are available from the corresponding author upon request.

## Supplementary Materials

Supplementary Materials Supplementary Material 1. Supplemental Table 1: fatty acid % composition of diets 2. Supplemental Table 2: effect of dietary lipids on fatty acid % composition of serum measured by gas-liquid chromatography following early intervention 3. Supplemental Table 3: effect of dietary lipids on fatty acid % composition of serum measured by gas-liquid chromatography following late intervention 4. Supplemental Table 4: effect of dietary Lipids on fatty acid % composition of the liver measured by gas-liquid chromatography following early intervention 5. Supplemental Table 5: effect of dietary lipids on fatty acid % composition of the liver measured by gas-liquid chromatography following late intervention 6. Supplemental Table 6: fatty acid unsaturation indices of serum and the liver following early intervention 7. Supplemental Table 7: fatty acid unsaturation indices of serum and the liver following late intervention 8. Supplemental Table 8: ratio of n-6 to n-3 fatty acids of the liver and serum measured by gas-liquid chromatography following early intervention 9. Supplemental Table 9: ratio of n-6 to n-3 fatty acids of the liver and serum measured by gas-liquid chromatography following late intervention 10. Supplemental Figure 1: vascular relaxation to acetylcholine (top) and calcitonin gene-related peptide (bottom) in epineurial arterioles of the sciatic nerve at the time of early intervention in high-fat-fed diabetic Sprague-Dawley rats.
