# Supplementary material for: Effect of Early and Late Interventions with Dietary Oils on Vascular and Neural Complications in a Type 2 Diabetic Rat Model
Source: J Diabetes Res. 2019 Aug 7;2019:5020465. doi: 10.1155/2019/5020465 (PMC6702827; doi:10.1155/2019/5020465)
Supplement: Supplementary Materials — Supplementary Material 1. Supplemental Table 1: fatty acid % composition of diets 2. Supplemental Table 2: effect of dietary lipids on fatty acid % composition of serum measured by gas-liquid chromatography following early intervention 3. Supplemental Table 3: effect of dietary lipids on fatty acid % composition of serum measured by gas-liquid chromatography following late intervention 4. Supplemental Table 4: effect of dietary Lipids on fatty acid % composition of the liver measured by gas-liquid chromatography following early intervention 5. Supplemental Table 5: effect of dietary lipids on fatty acid % composition of the liver measured by gas-liquid chromatography following late intervention 6. Supplemental Table 6: fatty acid unsaturation indices of serum and the liver following early intervention 7. Supplemental Table 7: fatty acid unsaturation indices of serum and the liver following late intervention 8. Supplemental Table 8: ratio of n-6 to n-3 fatty acids of the liver and serum measured by gas-liquid chromatography following early intervention 9. Supplemental Table 9: ratio of n-6 to n-3 fatty acids of the liver and serum measured by gas-liquid chromatography following late intervention 10. Supplemental Figure 1: vascular relaxation to acetylcholine (top) and calcitonin gene-related peptide (bottom) in epineurial arterioles of the sciatic nerve at the time of early intervention in high-fat-fed diabetic Sprague-Dawley rats. [file 5020465.f1.pdf]

Supplemental Table 1: Fatty Acid % Composition of Diets

Diet	16:0	18:0	18:1	18:2	18:3 (n-6)	18:3 (n-3)	20:5	22:6
Control	21 ± 3.0	7.3 ± 1.2	30.4 ± 4.0	31.7 ± 5.1	2.8 ± 0.6	< 1	< 1	< 1
High Fat	16.8 ± 2.2	10.8 ± 1.3	41.2 ± 5.6	26.9 ± 2.8	2.3 ± 0.3	< 1	< 1	< 1
Olive oil	14.9 ± 2.4	5.4 ± 0.7	49.7 ± 6.3	21.3 ± 3.0	2.0 ± 0.1	< 1	< 1	< 1
Safflower oil	12.3 ± 0.7	6.0 ± 0.5	24.9 ± 3.4	54.3 ± 4.3	< 1	< 1	< 1	< 1
Evening Primrose oil	11.6 ± 1.7	6.0 ± 0.7	20.7 ± 2.4	52.5 ± 4.0	5.4 ± 0.8	< 1	< 1	< 1
Flaxseed oil	11.1 ± 1.0	6.2 ± 1.0	27.0 ± 2.8	23.6 ± 3.4	< 1	31.0 ± 3.3	< 1	< 1
Menhaden oil	18.7 ± 0.9	7.1 ± 0.6	22.6 ± 2.6	16.0 ± 0.9	< 1	2.0 ± 0.1	9.5 ± 1.0	6.9 ± 0.4

Data are presented as the mean of three determinations ± S.E.M.

Supplemental Table 2: Effect of Dietary Lipids on Fatty Acid % Composition of Serum Measured by Gas-liquid Chromatography Following Early Intervention

Condition	16:0	18:0	18:1	18:2	18:3 (n-6)	18: 3 (n-3)	20:4	20:5	22:6
Control (3)	20.7± 0.4	18.4 ± 0.4	11.0 ± 0.4	19.6 ± 0.8	0.2 ± 0.1	0.1 ± 0.1	20.6 ± 0.8	0.7 ± 0.1	2.9 ± 0.3
Diabetic (3)	19.9 ± 0.6	13.5 ± 0.7[a]	9.5 ± 0.7	20.3 ± 0.9	0.2 ± 0.1	0.1 ± 0.1	20.2 ± 1.4	0.5 ± 0.1	4.1 ± 0.1
Diabetic + Olive oil (3)	21.0 ± 1.6	20.6 ± 1.1	16.1 ± 1.5[a,b]	19.6 ± 2.0	0.2 ± 0.1	0.2 ± 0.1	15.4 ± 1.2	0.4 ± 0.1	4.6 ± 0.6
Diabetic + Safflower oil (3)	19.1 ± 0.7	17.4 ± 0.8	8.0 ± 0.3	28.2 ± 1.2[a,b]	0.2 ± 0.1	0.2 ± 0.1	18.5 ± 1.0	0.3 ± 0.1	3.7 ± 0.3
Diabetic + Evening Primrose oil (3)	18.5 ± 0.5	20.8 ± 0.3	7.6 ± 0.3	22.6 ± 1.0	1.4 ± 0.2[a,b]	0.2 ± 0.1	18.6 ± 1.8	0.4 ± 0.1	2.6 ± 0.4
Diabetic + Flaxseed oil (3)	17.7 ± 1.0	16.8 ± 0.4	9.9 ± 0.4	25.1 ± 2.0	0.3 ± 0.1	4.5 ± 0.2[a,b]	11.2 ± 0.5[a,b]	1.9 ± 0.1[a,b]	5.2 ± 0.6[a]
Diabetic + Menhaden oil (3)	21.8 ± 0.5	15.0 ± 1.0	7.8 ± 1.1	18.7 ± 1.1	0.2 ± 0.1	0.4 ± 0.1	12.2 ± 1.3[a,b]	7.8 ± 0.3[a,b]	8.8 ± 0.9[a,b]

Data are presented as the mean ± S.E.M. a $P < 0.05$ compared to control rats; b $P < 0.05$ compared to diabetic rats. The number of animals in each group is shown in parenthesis.

Supplemental Table 3: Effect of Dietary Lipids on Fatty Acid % Composition of Serum Measured by Gas-liquid Chromatography Following Late Intervention

Condition	16:0	18:0	18:1	18:2	18:3 (n-6)	18: 3 (n-3)	20:4	20:5	22:6
Control (3)	21.2± 0.4	15.9 ± 0.6	9.4 ± 0.3	17.3 ± 1.2	0.2 ± 0.1	0.1 ± 0.1	14.6 ± 2.0	0.7 ± 0.1	3.6 ± 0.1
Diabetic (3)	19.7 ± 1.8	19.5 ± 1.1	10.6 ± 0.5	18.3 ± 2.1	0.3 ± 0.1	0.1 ± 0.1	16.6 ± 2.1	0.5 ± 0.1	3.9 ± 0.8
Diabetic + Olive oil (3)	18.9 ± 1.2	19.1 ± 1.5	12.8 ± 1.4	15.6 ± 1.4	0.2 ± 0.1	0.3 ± 0.1	17.9 ± 2.2	0.4 ± 0.1	4.1 ± 0.6
Diabetic + Safflower oil (3)	19.1 ± 0.5	20.4 ± 1.8	6.0 ± 1.1	23.9 ± 1.1	0.3 ± 0.1	0.2 ± 0.1	18.3 ± 1.6	0.3 ± 0.1	3.8 ± 0.4
Diabetic + Evening Primrose oil (3)	19.9 ± 1.0	20.3 ± 1.0	5.8 ± 1.0	24.6 ± 1.4	0.7 ± 0.1	0.2 ± 0.1	19.6 ± 1.4	0.4 ± 0.1	3.3 ± 0.7
Diabetic + Flaxseed oil (3)	17.5 ± 0.8	21.8 ± 1.2	6.9 ± 1.7	21.8 ± 1.4	0.2 ± 0.1	5.2 ± 1.5[a,b]	13.2 ± 1.6	1.5 ± 0.1[a,b]	4.2 ± 0.2
Diabetic + Menhaden oil (3)	23.4 ± 0.5	15.0 ± 1.2	10.5 ± 1.3	19.5 ± 0.8	ND	0.7 ± 0.1	8.0 ± 0.4[a,b]	7.4 ± 0.2[a,b]	7.6 ± 0.5[a,b]

Data are presented as the mean ± S.E.M. ND: not detected. a P < 0.05 compared to control rats; b P < 0.05 compared to diabetic rats. The number of animals in each group is shown in parenthesis.

Supplemental Table 4: Effect of Dietary Lipids on Fatty Acid % Composition of Liver Measured by Gas-liquid Chromatography Following Early Intervention

Condition	16:0	18:0	18:1	18:2	18:3 (n-6)	18:3 (n-3)	20:4	20:5	22:6
Control (3)	18.6 ± 1.7	13.6 ± 1.7	8.5 ± 0.2	10.5 ± 1.1	0.8 ± 0.3	0.1 ± 0.1	18.8 ± 2.0	1.7 ± 0.4	6.5 ± 0.4
Diabetic (3)	16.3 ± 1.6	18.9 ± 2.1	10.8 ± 1.6	12.0 ± 1.2	1.2 ± 0.1	0.2 ± 0.1	17.4 ± 0.4	2.1 ± 1.0	6.7 ± 0.3
Diabetic + Olive oil (3)	16.4 ± 0.2	20.8 ± 1.7[a]	13.7 ± 1.9[a]	13.3 ± 1.7	0.7 ± 0.1	0.3 ± 0.1	19.0 ± 1.4	1.1 ± 0.4	6.7 ± 0.3
Diabetic + Safflower oil (3)	15.9 ± 1.1	20.9 ± 0.7[a]	7.3 ± 0.8	20.1 ± 2.5[a]	0.9 ± 0.1	0.3 ± 0.1	19.3 ± 0.3	1.0 ± 0.3	6.5 ± 0.5
Diabetic + Evening Primrose oil (3)	15.4 ± 1.1	22.8 ± 0.7	5.2 ± 0.3[b]	16.1 ± 2.5	1.2 ± 0.1	0.2 ± 0.2	21.2 ± 1.1	0.9 ± 0.6	5.3 ± 0.3
Diabetic + Flaxseed oil (3)	14.5 ± 1.1	19.6 ± 1.0	9.0 ± 0.6	17.3 ± 2.3	0.4 ± 0.1	4.4 ± 0.5[a,b]	14.3 ± 0.7	3.1 ± 0.4	6.2 ± 1.2
Diabetic + Menhaden oil (3)	20.0 ± 0.4	20.5 ± 1.7	7.7 ± 0.7	13.5 ± 1.0	0.3 ± 0.1	0.2 ± 0.1	12.1 ± 0.7[a,b]	4.8 ± 0.6[a]	14.4 ± 0.2[a,b]

Data are presented as the mean ± S.E.M. a $P < 0.05$ compared to control rats; b $P < 0.05$ compared to diabetic rats. The number of animals in each group is shown in parenthesis.

Supplemental Table 5: Effect of Dietary Lipids on Fatty Acid % Composition of Liver Measured by Gas-liquid Chromatography Following Late Intervention

Condition	16:0	18:0	18:1	18:2	18:3 (n-6)	18:3 (n-3)	20:4	20:5	22:6
Control (3)	21.3 ± 2.8	17.0 ± 2.4	9.2 ± 0.7	12.2 ± 0.2	0.4 ± 0.1	0.2 ± 0.1	20.3 ± 0.8	0.7 ± 0.1	5.2 ± 0.6
Diabetic (3)	18.7 ± 0.7	20.3 ± 0.2	12.1 ± 0.4[a]	14.5 ± 1.4	0.3 ± 0.1	0.1 ± 0.1	20.0 ± 0.7	0.7 ± 0.1	6.9 ± 1.3
Diabetic + Olive oil (3)	15.0 ± 0.9	17.2 ± 1.1	11.1 ± 1.0	9.6 ± 0.9	0.2 ± 0.1	0.2 ± 0.1	18.7 ± 1.0	1.0 ± 0.1	7.8 ± 0.6
Diabetic + Safflower oil (3)	19.0 ± 1.7	19.7 ± 2.3	7.7 ± 0.3[b]	20.2 ± 1.6[a,b]	0.4 ± 0.1	0.3 ± 0.1	18.0 ± 1.2	0.5 ± 0.2	6.6 ± 0.5
Diabetic + Evening Primrose oil (3)	16.4 ± 0.8	19.8 ± 1.4	7.0 ± 0.3[b]	18.3 ± 1.2[a]	0.3 ± 0.1	0.4 ± 0.2	20.7 ± 0.2	0.8 ± 0.1	6.0 ± 1.1
Diabetic + Flaxseed oil (3)	17.8 ± 0.2	24.1 ± 1.0	5.6 ± 0.4[a,b]	17.9 ± 0.2[a]	0.3 ± 0.1	3.8 ± 0.6[a,b]	16.0 ± 0.5[a,b]	2.4 ± 0.1[a,b]	7.7 ± 0.1
Diabetic + Menhaden oil (3)	20.5 ± 1.4	16.1 ± 0.6	9.5 ± 0.7	14.4 ± 1.4	0.4 ± 0.1	0.5 ± 0.1	10.0 ± 0.2[a,b]	5.9 ± 0.6[a,b]	13.7 ± 0.9[a,b]

Data are presented as the mean ± S.E.M. a $P < 0.05$ compared to control rats; b $P < 0.05$ compared to diabetic rats. The number of animals in each group is shown in parenthesis.

Supplemental Table 6: Fatty Acid Unsaturation Indices of Serum and Liver Following Early Intervention

Condition	Serum	Liver
Control	1.69 ± 0.02	1.74 ± 0.03
Diabetic	1.63 ± 0.04	1.78 ± 0.07
Diabetic + Olive oil	1.50 ± 0.06	1.73 ± 0.05
Diabetic + Safflower oil	1.54 ± 0.02	1.77 ± 0.04
Diabetic + Evening Primrose oil	1.54 ± 0.06	1.77 ± 0.05
Diabetic + Flaxseed oil	1.62 ± 0.05	1.78 ± 0.09
Diabetic + Menhaden oil	1.89 ± 0.04[b]	2.07 ± 0.01[a,b]

Data are presented as the mean ± S.E.M. a $P < 0.05$ compared to control rats; b $P < 0.05$ compared to diabetic rats.

Supplemental Table 7: Fatty Acid Unsaturation Indices of Serum and Liver Following Late Intervention

Condition	Serum	Liver
Control	1.67 ± 0.05	1.60 ± 0.03
Diabetic	1.52 ± 0.06	1.76 ± 0.04
Diabetic + Olive oil	1.60 ± 0.03	1.76 ± 0.03
Diabetic + Safflower oil	1.59 ± 0.02	1.73 ± 0.05
Diabetic + Evening Primrose oil	1.60 ± 0.04	1.75 ± 0.05
Diabetic + Flaxseed oil	1.62 ± 0.03	1.87 ± 0.01[a]
Diabetic + Menhaden oil	1.75 ± 0.04[b]	2.03 ± 0.03[a,b]

Data are presented as the mean ± S.E.M. a $P < 0.05$ compared to control rats; b $P < 0.05$ compared to diabetic rats.

Supplemental Table 8: Ratio of n-6 to n-3 Fatty Acids of Liver and Serum Measured by Gas-liquid Chromatography Following Early Intervention

Condition	Serum	Liver
Control	7.3 ± 0.4	3.6 ± 0.6
Diabetic	7.8 ± 1.0	3.4 ± 0.6
Diabetic + Olive oil	7.0 ± 0.7	4.1 ± 0.4
Diabetic + Safflower oil	9.7 ± 1.1	5.0 ± 0.7
Diabetic + Evening Primrose oil	11.5 ± 1.6	6.6 ± 0.5
Diabetic + Flaxseed oil	3.2 ± 0.4[a]	2.3 ± 0.1
Diabetic + Menhaden oil	1.8 ± 0.2[a,b]	1.3 ± 0.1[a,b]

Data are presented as the mean ± S.E.M. a $p < 0.05$ compared to control rats; b $p < 0.05$ compared to diabetic rats.

Supplemental Table 9: Ratio of n-6 to n-3 Fatty Acids of Liver and Serum Measured by Gas-liquid Chromatography Following Late Intervention

Condition	Serum	Liver
Control	9.1 ± 0.6	5.2 ± 1.0
Diabetic	8.4 ± 1.4	5.2 ± 1.0
Diabetic + Olive oil	7.7 ± 0.7	2.9 ± 0.4
Diabetic + Safflower oil	10.6 ± 1.3	5.3 ± 0.4
Diabetic + Evening Primrose oil	12.9 ± 2.2	5.9 ± 0.7
Diabetic + Flaxseed oil	3.4 ± 0.7[a]	2.4 ± 0.1
Diabetic + Menhaden oil	1.8 ± 0.1[a,b]	1.2 ± 0.1[a,b]

Data are presented as the mean ± S.E.M. a $p < 0.05$ compared to control rats; b $p < 0.05$ compared to diabetic rats.

Supplemental Figure 1

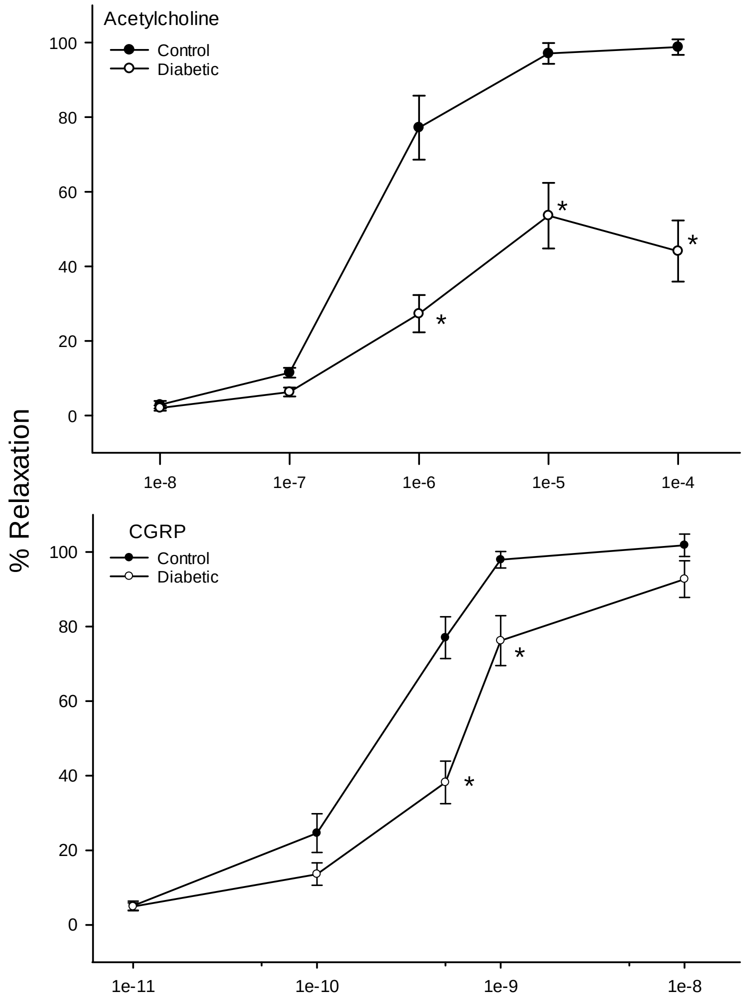

Vascular relaxation to acetylcholine (top) and calcitonin gene-related peptide (bottom) in epineurial arterioles of the sciatic nerve at the time of early intervention in high fat fed diabetic Sprague-Dawley rats; $p < 0.05$ compared to control rats.
